# Determination of the electronic transport in type separated carbon nanotubes thin films doped with gold nanocrystals

**DOI:** 10.1038/s41598-021-96307-6

**Published:** 2021-08-17

**Authors:** M. Świniarski, A. Dużyńska, A. P. Gertych, K. Czerniak-Łosiewicz, J. Judek, M. Zdrojek

**Affiliations:** Faculty of Physics, Warsaw University of Technology, Koszykowa 75, 00-662 Warszawa, Poland

**Keywords:** Electronic properties and materials, Surfaces, interfaces and thin films

## Abstract

We report a systematic theoretical and experimental investigation on the electronic transport evolution in metallic and semiconducting carbon nanotubes thin films enriched by gold nanocrystals. We used an ultra-clean production method of both types of single-walled carbon nanotube thin films with/without gold nanocrystals, which were uniformly dispersed in the whole volume of the thin films, causing a modification of the doping level of the films (verified by Raman spectroscopy). We propose a modification of the electronic transport model with the additional high-temperature features that allow us to interpret the transport within a broader temperature range and that are related to the conductivity type of carbon nanotubes. Moreover, we demonstrate, that the proposed model is also working for thin films with the addition of gold nanocrystals, and only a change of the conductivity level of our samples is observed caused by modification of potential barriers between carbon nanotubes. We also find unusual behavior of doped metallic carbon nanotube thin film, which lowers its conductivity due to doping.

## Introduction

The utilization of carbon nanotubes (CNTs) in thin-film production raised in the last couple of years, because of possible industrial applicability. The CNTs networks might significantly impact modern electronics and photonics combining the optical and electrical properties of one dimensional objects with the applicability of large-area thin films^1,2^. The devices made from CNT thin films such as FET transistors^3^, sensors^4^, and transparent electrodes^5,6^ have been widely reported in recent years, but they were mostly made of a mixture of 1/3 metallic and 2/3 semiconducting carbon nanotubes^7^. When the type-separated carbon nanotubes started to be investigated^7–9^, it was shown that the material's electrical properties are strongly related to the type of nanotubes^10–12^. The carbon nanotube thin film made of metallic carbon nanotubes show higher electrical conductivity than semiconducting films and the gate responses are different with respect to carbon nanotube type^13–16^, allowing to use it accordingly to application requirements^17^. An additional factor that influences conductivity in such materials is the structural arrangement of CNTs inside the system. Such metamaterials are built of a large number of randomly distributed interconnections between the tubes, which are seen as an additional energy gap for electrons that result in a decrease in conductivity. The films conductivity could be further controlled by nanotube surface modifications^18^ i.e. Au nanoparticles (NPs)^19–23^, SOCl_2_^24–26^, PABS^27^, and many others^28,29^. The functionalized carbon nanotubes show changes in conductivity either increase (Au, SOCl_2_)^22,24^ or decrease (CdTe QD, aniline)^24,30^. Thus, one can modify the conductivity of the material by controlled doping of carbon nanotubes. The utilization of electronic devices, including those based on CNT films, often requires operation in a wide temperature range (− 200 to 200 ℃). Since doping could set our conductivity to the desired value, the crucial issue is to know how it influences the electronic transport, especially in a wide temperature range.

The literature provides a vast range of research devoted to electronic transport in most of the available types of CNTs systems: non-separated, type-separated, thick (> 100 nm), thin (< 100 nm), doped and undoped^24,28,31,32^. Also, various theoretical models are employed: Fluctuation Induced Tunneling (FIT)^33^, 3 and 2-dimensional Variable Range Hopping (3D, 2D-VRH)^34^ and modified VRH (ES-VRH)^35^. However, most of the studies are focused on electronic transport in carbon nanotubes networks, without consideration of doping effects. On the other hand, when the doping effects are taken into account, they are considered usually in the view of how conductivity or transmittance are affected^22,23,29^. No doping influence on the transport model has been considered before and also very little attention was given to the influence of doping on the temperature-dependent conductivity of CNT films^20,25,26^.

In this work, we present the systematic study on electronic transport of the type-separated carbon nanotubes thin films in a wide temperature range (77–450 K). We used an ultra-clean production method of metallic (mCNT) and semiconducting (sCNT) single-walled carbon nanotube thin films with and without gold nanocrystals (Au NCs). We show, that the electronic transport models in higher temperatures must be modified with the additional high-temperature features that are related to the conductivity type of carbon nanotubes. Moreover, we show that the implementation of AuNCs in the whole volume of carbon nanotube thin film does not affect the transport model, but only changes our samples' conductivity level by changing potential barriers between carbon nanotubes. The influence of p-type doping has been proved by statistical measurements with Raman spectroscopy, where spectra are sensitive on charge carrier concentrations. We also find unusual behavior of doped metallic carbon nanotube thin film, which lowers its conductivity as a result of doping.

## Materials and methods

### Carbon nanotube thin films preparation

**Figure 1:**
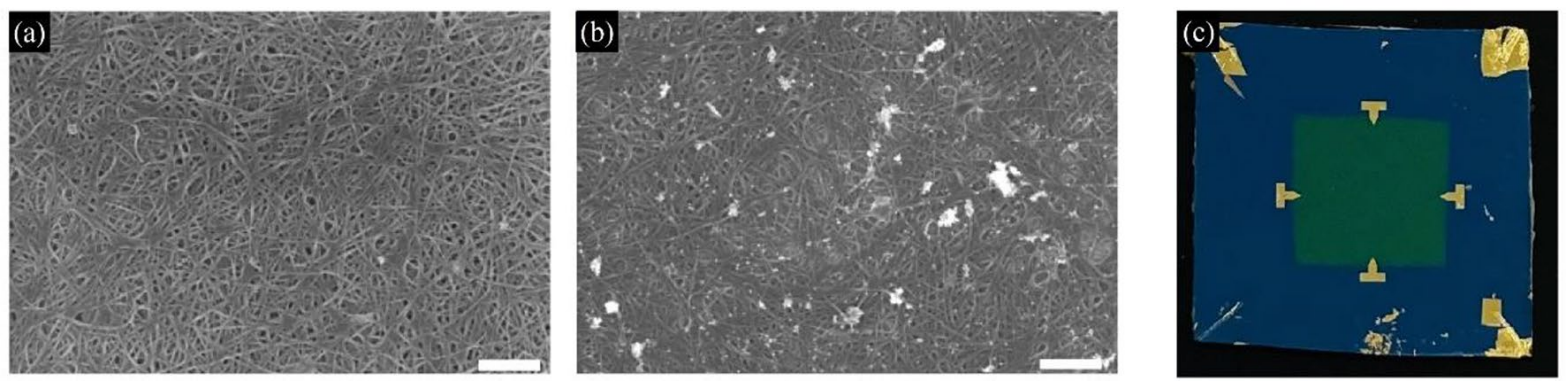
The SEM analysis pictures taken from the mCNT samples without (**a**) and with (**b**) gold nanocrystals (the white areas). The scale bars are 400 nm. (**c**) Photograph of the representative sample of thin carbon nanotube film with four contacts located at the edges of the film.

To produce thin films, we used commercially available of type separated carbon nanotubes purchased from NanoIntegris Inc. The ultra-clean preparation of thin films requires proper dispersion of carbon nanotubes in a solution of deionized water and sodium dodecyl sulfate (SDS), which reduces surface tension in a further application into a vacuum filtration process. The filtered and dried thin films of CNTs were deposited on Si/SiO_2_ substrate. Carefully prepared vacuum filtration of carbon nanotube solution provides a thin layer (50 nm) of uniformly dispersed carbon nanotubes^36^, which is presented in Fig. 1a. Samples with gold nanocrystals were prepared with an additional step of dispersion of the Au NCs into a previously prepared solution of carbon nanotubes, which further was applied to the vacuum filtration process (for more details please see “Supplementary Information”). This approach ensures quite uniform dispersion of gold nanocrystals into a whole volume of carbon nanotube thin film (see Fig. 1b and Fig. S2). For our investigations, we have prepared two sets of samples—pure metallic (mCNT) and pure semiconducting (sCNT) carbon nanotubes and two sets of samples with gold nanocrystals (mCNT + Au and sCNT + Au).

### Electrical characterization

Next, the four 1 gold contacts (100 nm thick) were attached to the film using a standard e-beam lithography process followed by a lift-off step (see Fig. 1c). For electrical measurements samples were put into cryostat (Oxford MicrostatHe2) and using the set of Keithley 2450 SMU, Keithley 7001 switch unit equipped with Hall card, and National Instruments PC-4628, the four contact measurements were performed. The van der Pauw four contact method ensures that contact resistance is negligible in our case. The temperature electrical measurements were performed in vacuum conditions in the following scheme: (1) 450 K annealing for humidity evaporation for at least 12 h, (2) 450 K → 70 K → 450 K → 300 K. The data has been characterized by negligible hysteresis, which exclude the influence of the de-doping effect^26,37^ (see “Supplementary Information”, Fig. S5).

### Structural characterization

The structural parameters were evaluated with statistical Raman measurements using the Renishaw InVia spectrometer. The Raman mapping was performed on an area of 40 μm × 40 μm with a collecting step of 4 μm (100 points) using a laser wavelength of 514 nm. To avoid heating effects we have used low laser power of 0.35 mW, which has been additionally defocused, resulting in laser spot size of 4 μm, as for our previous invesitgation^38–40^. We performed statistical analysis of 100 Raman spectra for each sample, and fit the Lorentzian or BWF function to obtained set of parameters: position of G^+^, G^−^, D and 2D modes, which further has been used for correlation analysis.

## Results and discussion

### Doping effect on the structural parameters

**Figure 2:**
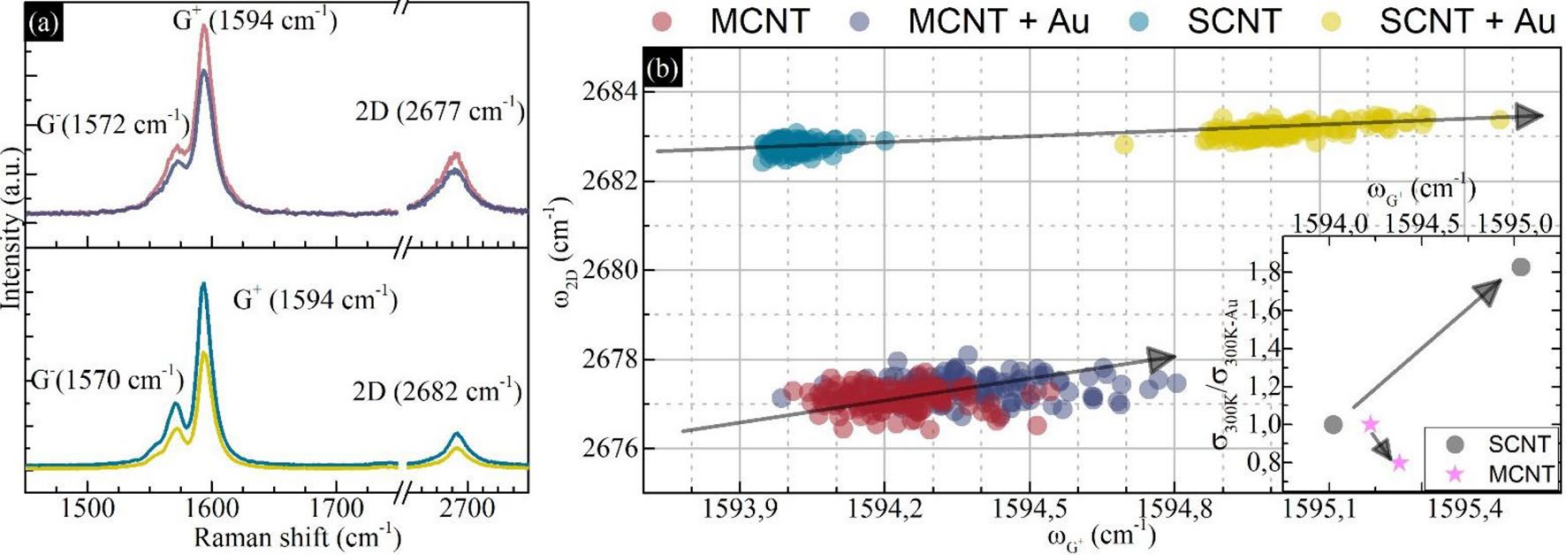
(**a**) The representative spectra collected from mCNT, mCNT + Au, sCNT and sCNT + Au. (**b**) Correlation of 2D mode and G^+^ mode positions with an inset consisting of normalized changes of conductivity versus G^+^ mode position.

The quality of prepared carbon nanotube thin films is crucial in terms of interpretation of the electrical transport. One of the most valuable methods of investigating the low dimensional materials quality is Raman spectroscopy. Figure 2a presents the comparison of representative spectra collected for mCNT and sCNT. The difference between the types of carbon nanotubes is seen, in the G mode, which in both cases are built with two main components: one peaked at ~ 1590 cm^−1^ (G^+^), and the next peaked at ~ 1570 cm^−1^ (G^−^). The metallic carbon nanotubes are characterized by lower splitting between the ω_G+_ and ω_G−_ than semiconducting. In our case, the differences are 22 cm^−1^ for metallic tubes and 25 cm^−1^ for semiconducting tubes. The lineshape for metallic G^−^ mode should be well described by Breigt–Winger–Fano (BWF) function, in contrast to the Lorentzian shape for semiconducting tubes^41^ (see also Fig. S1 in “Supplementary Information”). Moreover, the 2D mode position should be lower for metallic carbon nanotubes in comparison to semiconducting, which is in good agreement with our observations (ω_2D_ = 2677 cm^−1^ for metallic and ω_2D_ = 2682 cm^−1^ for semiconducting). Additional, the quality of the carbon nanotubes could be determined by the ratio of the intensities of D (I_D_) mode and G^+^ (I_G_) mode^41^, where the lower value refers to better quality, and in our case, the I_D_ is negligible (see Fig. S3), which indicates high-quality carbon nanotubes in our thin films.

The Raman spectroscopy is also sensitive to changes in carriers concentration in carbon nanotubes caused by doping^41,42^. Firstly, for the doped samples mCNT + Au and sCNT + Au we observed a decrease in the intensity (Fig. 2a), which is probably caused by lowering the number of electrons that are involved in the Raman scattering process by an acceptor (Au^3+^) doping^41^. Secondly, the concentration-sensitive modes G^+^ and 2D, which change their positions as a result of doping-induced concentration changes. The ω_2D_ redshifts are identified as an acceptor doping, while ω_G+_ shift indicates doping itself^43^. Figure 2b presents the statistical correlation of position of doping sensitive modes ω_2D_ (ω_G+_). In both cases mCNT + Au and sCNT + Au we observe position shifts, which indicates the acceptor doping by Au^3+^ ions. The shifts for metallic carbon nanotube thin film (Δω_G+_ = 0.14 cm^−1^, Δω_2D_ = 0.24 cm^−1^) are lower compare to shift for semiconducting thin film (Δω_G+_ = 0.33 cm^−1^, Δω_2D_ = 0.98 cm^−1^) (see Fig. S2), which are reflected in the relative changes in conduction. The inset in Fig. 2b shows normalized conductivity at 300 K (σ_300K_/σ_300K+Au_) as a function of ω_G+_. For sCNT samples, the conductivity increased by 83%, as a result of an increase of major charge carriers concentration by acceptor doping. For mCNT conductivity decreased by 21%, which is rather unusual behavior for the metallic carbon nanotubes. This suggests, that the initial doping of mCNT was n-type, and after injection of an acceptor dopant, the concentration of major charge carriers is lowered. The relative changes of conduction correlate with changes of concentration, which proves that observed structural changes visible in Raman spectra are the result of doping by Au NCs. The structural investigations also agree with previous investigations, where sCNT is more sensitive to charge carrier concentration than mCNT^26,28^.

### Electrical transport investigation

The temperature dependence of the conductivity of the carbon nanotube thin films with its modifications by doping leads to a better understanding of the nature of the electrical transport in carbon nanotube thin films. The carbon nanotubes films are highly disordered systems, which could be modeled as randomly distributed conducting wires—this crucial fact has been noticed by Alan B. Kaiser by comparing CNTs thin films to conducting polymers^44^. The electronic transport in our samples has been tested by four common theoretical models: Fluctuation Induced Tunneling (FIT)^33^, 3- and 2-dimensional Variable Range Hopping (3D-, 2D-VRH)^34^, and modified VRH by Efros–Shklovskii^35^, which takes into account Coulomb interactions (ES-VRH). The determination of the electronic transport model is challenging, because of two issues: (1) the simple models do not follow the experimental data within the whole temperature range (they fail in higher temperatures range), (2) in certain temperature range all model gives reasonable fitting results (see Fig. S3 in the “Supplementary Information”), even though much different physics is found behind each one. Here, we show some new insight.

**Figure 3:**
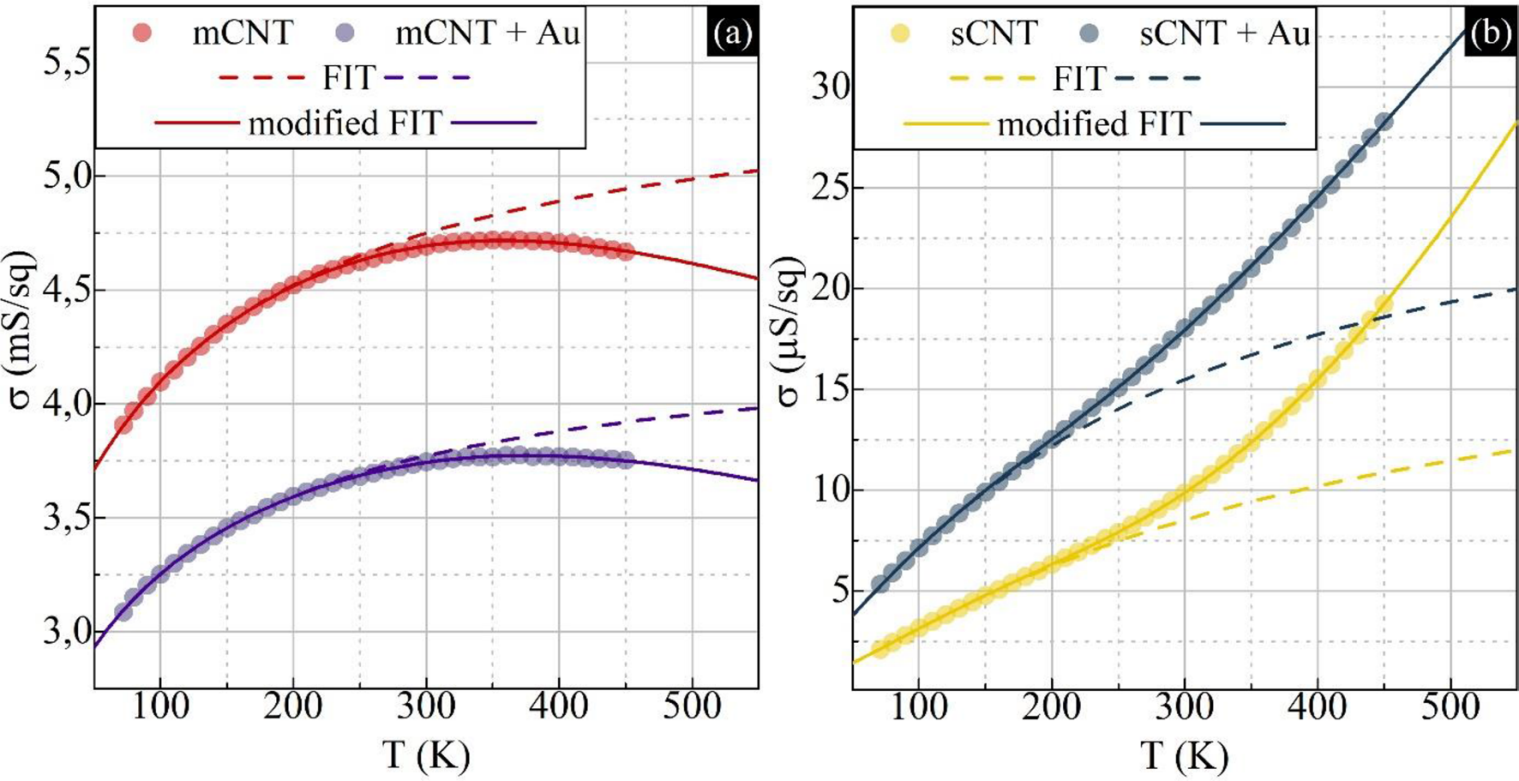
The conductivity dependence from temperature for electronic transport evaluation. (**a**) mCNT and mCNT + Au comparison with fitted Fluctuation Induced Tunneling model with (solid line) and without (dashed line) high-temperature components. (**b**) sCNT and sCNT + Au comparison with the fitted FIT model with (solid line) and without (dashed line) high-temperature components. All collected data has been characterized by uncertainty less than 1% (see “Supplementary Information”).

Figure 3 presents how the electrical conductivity evolves with temperature for both types of carbon nanotube thin film. The data has been characterized by small uncertainty, which is not visible on the Fig. 3. The uncertainty has been discussed in “Supplementary Information”, section *The data collection and error discussion*. Since all tested models should produce an increase of the conductivity in a wide range of the temperature as it is presented as a dashed line in Fig. 3 (for FIT model) and in Fig. S3 (for VRH models). We noticed an interesting behavior especially for the mCNT and mCNT + Au data, where the slope (∂σ(T)/∂T) changes its sign above 260 K (Fig. 3a). A similar phenomenon was measured in non-separated Buckypapers^45–47^, where the high-temperature factor was interpreted as a characteristic phonon scattering in 1D conductor^11,12^ or as a result of the concentric expansion of the tubes (radial breathing), which constantly change the distance between tubes and reduce the tunneling probability^41,48^. This effect could be also related with the de-doping effect^26,37^, but in our case we excluded this by proper annealing before measurement (see Fig. S5). Moreover, we also noticed a change of slope for sCNT and sCNT + Au, which were previously documented, up to date, only in the semiconducting ultra-thin film (0.8–2 nm) by Itkis et.al.^31^ and interpreted as a high-temperature band-like transport. To the best of our knowledge, there is no report where such behavior in 50 nm pure carbon nanotube films was observed. Here, an additional high-temperature factor has been implemented in order to fully determinate the transport model. The formula with high-temperature components are described below:

1 $${\upsigma }^{-1}\left(T\right)=\rho (T)={\left(\text{A exp}\left[\frac{{\text{T}}_{\text{t}}}{T+{\text{T}}_{\text{s}}}\right]+\text{B exp}\left[\pm \frac{{E}_{a}}{{k}_{B}T}\right]\right)}^{-1},$$

where A and B are preexponential factors (A, B = f_A,B_ ρ_A,B_, where f is geometrical factor^44^). Equation (1) presents modified Fluctuation Induced Tunneling (modified FIT), where T_t_ is related to barrier height for electrons, T_S_ is the temperature below which elastic scattering dominates^33^. The “E_a_” refers to two additional formulas, “− E_a_” for phonon scattering (1D transport along CNT) in mCNT and “+ E_a_” for band-like transport in sCNT.

The metallic carbon nanotubes thin films are rare in literature in the scope of the electronic transport investigations, but it seems that the simple Fluctuation Induced Tunneling (in Eq. (1), B = 0) is the predominant transport model reported in metallic carbon nanotube Buckypaper^26^ and simulated thin film^14^. For the semiconducting carbon nanotube thin films, the situation is more complicated. One can find the simple FIT model for ultra-thin film^31^, 3D-VRH for drop-casted film^49^, and ES-VRH for semiconducting CNT Buckypaper^13^. Thus, the literature information might be misleading in proper transport model determination. To show this, we analyze how the ratio of the experimental data and theoretical fit changes in the given temperature range. All different models have been fitted and compared (Fig. S3), which leads us to conclude that the Fluctuation Induced Tunneling with the addition of high-temperature components (solid line in Fig. 3) is the most suitable transport model in mCNT and sCNT samples.

Interestingly, the implementation of the additional nanocrystals in the whole volume of the investigated thin films did not change the electronic transport model of our samples. In Fig. 3 and Fig. S3, we have noticed that the predominant transport model for mCNT + Au and sCNT + Au is still FIT, but the conductivity is different (Table 1).

**Table 1:** The comparison of the conductivity changes at 300 K and FIT model parameters.

Sample	σ (mS/sq) at 300 K	Modified-FIT (meV)		
		T_t_	T_S_	E_a_
mCNT	4.69	5.0	8.4	121
mCNT + Au	3.75	4.6	7.5	135
sCNT	0.01	26.8	5.8	143
sCNT + Au	0.02	19.2	5.2	106

Table 1 presents energies obtained from fitting the FIT model to our data. The mCNT + Au is characterized by lower conductivity, which we previously connected with the differences of the initial doping of mCNT (n-type) (Fig. 2b inset). We observe that T_t_ energy is slightly decreased in mCNT + Au, which could indicate that the potential barriers between tubes are lower. But the E_a_, which in the case of mCNT describes the transport limiting factor due to acoustic phonon scattering or reducing tunneling probability because of the radial breathing of the tube. The activation energy is higher after doping, which suggests that an additional mass on the tubes could increase the energy, which limits the conductivity. The sCNT + Au is characterized by higher conductivity, which is related to the initial doping of the tubes as p-type. While the relative changes of conductivity are higher for sCNT + Au (Fig. 2b inset), we also observe a higher change of T_t_ energy from 26.8 to 19.2 meV, which suggest lowering the potential barriers between the tubes, and the activation energy E_a_ is decreased indicating that additional acceptors shift the Fermi level toward valence band, which results in higher conductivity. Thus, the additional factor should be related to the intrinsic nature of semiconducting carbon nanotube. In our case, the average diameter of used semiconducting tubes is 1.5 nm, which should correspond to an energy gap of 0.6 eV, but our high-temperature factor indicates that the energy gap is around 0.3 eV. The difference is probably caused by the complexity of the thin film, where transport is ruled mostly by the connection between the carbon nanotubes.

## Conclusions

We showed that the electronic transport in type-separated carbon nanotubes can be described by a modified Fluctuation Induced Tunneling model completed with the high-temperature factors, which originates in the nature of the carbon nanotube type. The metallic carbon nanotube thin films are characterized by the peak of the conductance, due to scattering involving acoustic phonon or concentric expansion of the tubes. The semiconducting carbon nanotubes are characterized by a more rapid increase of conductivity in higher temperatures due to the thermal activation of charge carriers from the valence to the conduction band. The doping with gold nanocrystals results only in a change of conductivity not affecting the transport model, which is important in the view of future utilization of carbon nanotube devices. The predictable (stable) conductivity can be modified by proper volume doping, which allows us to set the conductivity to the required level and know how it behaves with temperature.

## Supplementary Information

Supplementary Information.

## Data Availability

The data that support the findings of this study are available from the corresponding author M.S., upon reasonable request.
