# Author Correction: Determination of the electronic transport in type separated carbon nanotubes thin films doped with gold nanocrystals

**DOI:** 10.1038/s41598-021-00823-4

**Published:** 2021-10-21

**Authors:** M. Świniarski, A. Dużyńska, A. P. Gertych, K. Czerniak-Łosiewicz, J. Judek, M. Zdrojek

**Affiliations:** Faculty of Physics, Warsaw University of Technology, Koszykowa 75, 00-662 Warszawa, Poland

Correction to: *Scientific Reports* 10.1038/s41598-021-96307-6, published online 17 August 2021

The original version of this Article contained an error in the Results and discussion, under the subheading ‘Electrical transport investigation’,

“The electronic transport in our samples has been tested by four common theoretical models: Fluctuation Induced Tunneling (FIT)^33^, 3- and 2-dimensional Variable Range Hopping (3D-, 2D-VRH)^34^, and modified VRH by Efros–Shlivoski^35^, which takes into account Coulomb interactions (ES-VRH).”

now reads:

“The electronic transport in our samples has been tested by four common theoretical models: Fluctuation Induced Tunneling (FIT)^33^, 3- and 2-dimensional Variable Range Hopping (3D-, 2D-VRH)^34^, and modified VRH by Efros–Shklovskii^35^, which takes into account Coulomb interactions (ES-VRH).”

The original Article has been corrected.

