# Supplementary material for: Determination of the electronic transport in type separated carbon nanotubes thin films doped with gold nanocrystals
Source: Sci Rep. 2021 Aug 17;11:16690. doi: 10.1038/s41598-021-96307-6 (PMC8371105; doi:10.1038/s41598-021-96307-6)
Supplement: Supplementary file 1 — Supplementary Information. [file 41598_2021_96307_MOESM1_ESM.docx]

# Supplementary

**Determination of the electronic transport in type separated carbon nanotubes thin films doped with gold nanocrystals**

M. Świniarski^1*^, A. Dużyńska^1^, A. P. Gertych^1^, K. Czerniak – Łosiewicz^1^, J. Judek^1^, M. Zdrojek^1^

*^1^Faculty of Physics, Warsaw University of Technology, Koszykowa 75, 00-662 Warszawa, Poland*

**Corresponding author: michal.swiniarski@pw.edu.pl*

## **Carbon nanotube thin film fabrication**

The single wall carbon nanotube thin films were produced using vacuum filtration process^1^. First, a suspension of carbon nanotubes in a water surfactant solution was prepared. Surfactant particles stick to the CNT walls and prevent to aggregation of nanotubes in liquid. Dry nanotubes from Nanointegris (99% purity, mean tube diameter ~1.4 nm, mean length ~0.5 µm) were added to a 1% water solution of sodium dodecyl sulfate (SDS) to achieve 0.01 mg/ml nanotube concentration. Next, the suspension was mixed using a bath sonicator (Elmasonic P 30H, 37kHz frequency, effective power 100W) for 3 hours. The process was performed using ice bath sonication and relatively low sonication power to prevent overheating of the mixture (temperature > 60^o^C negatively affects the surfactant properties). After sonication, the suspension was centrifuged at 12 000 rpm for 10 min to remove the unseparated nanotubes. An appropriate amount of as-prepared carbon nanotube suspension (6 ml) has been vacuum filtered onto a Mixed Cellulose Ester (MCE) membrane purchased from Millipore (0.025 um pore size, 25 mm diameter) to achieve the thickness of 50 nm. Film thickness was controlled by the atomic force microscopy measurements (in the way described in our previous works ^1,2^). To ensure that SDS was rinsed out, we apply plenty of deionized through the thin film in the vacuum filtration system. The CNT/MCE film has been cut into 5 x 5 mm squares. Each square has been placed into acetone to dissolve the cellulose filter. The process was repeated through several baths to ensure that the MCE filter has been dissolved. Next, the acetone was replaced by the isopropanol/water solution with the ratio of 1:1 (to lower the surface tension), and the CNT film was picked up onto silicon substrate and gently dried by the nitrogen stream. To make sure that all contamination has been removed (MCE residues), we performed additional annealing on hot-plate at 250 C for 2 h. We have not noticed any structural changes in the thin film after annealing (Figure S1).

The samples enriched with gold nanocrystals have been produced with one additional step included in the above-mentioned process. The nanoparticles of gold purchased from MKnano (Au dots, size: 20 nm, stabilized with HAuCl_4_ in a concentration of 7.0 x 10^11^ particles/ml), have been introduced into CNT water dispersion. In detail, to 6 ml of carbon nanotubes’ suspension was added 1ml of gold nanocrystals’ dispersion (mCNT+Au). To ensure that the gold is properly dispersed into the solution of the CNT, we treated the solution with a sonication procedure (10 min, 35 ^o^C). What is important, additional sonication and temperature of 35^o^C do not affect the structure of carbon nanotubes and nanoparticles but improve adhesion between them, nanotube de-bundling is also not observed (confirmed by the SEM and Raman measurements). SEM images do not show differences between nanotube arrangements with and without gold nanocrystals. Next, the fabrication procedure followed the above-mentioned recipe. During the annealing process (250^o^C for 2 h) to decontaminate our samples from MCE residues and improve adhesion to the SiO_2_/Si substrate, the gold nanodots have created nanocrystals (see Figure S2). Moreover, the annealing at a temperature of 250 C should remove most of the chloride anions^3^. This should also cause the de-doping effect, which reduces the effect of ([AuCl_4_]^-^) influence, but it also stabilizes the doping effect itself. In our investigations, the conductivity changes are reversible and stable in a wide range of temperatures, which is important in any application.

| sCNT before annealing | sCNT after annealing |
| --- | --- |
| **** |  |

Figure S1 The SEM images of the sCNT thin films before and after annealing at 250 ^o^C for 2 hours.

| sCNT + Au before annealing | After 250 C annealing for 2 h   |
| --- | --- |

Figure S2 The SEM images of the gold nanodots doped sample before (left panel) and after annealing procedure at 250 ^o^C for 2h. The gold nanodots have created nanocrystals in places where there was an agglomerate with more than one nanodot.

## **The Lineshape analysis and D-mode**

| 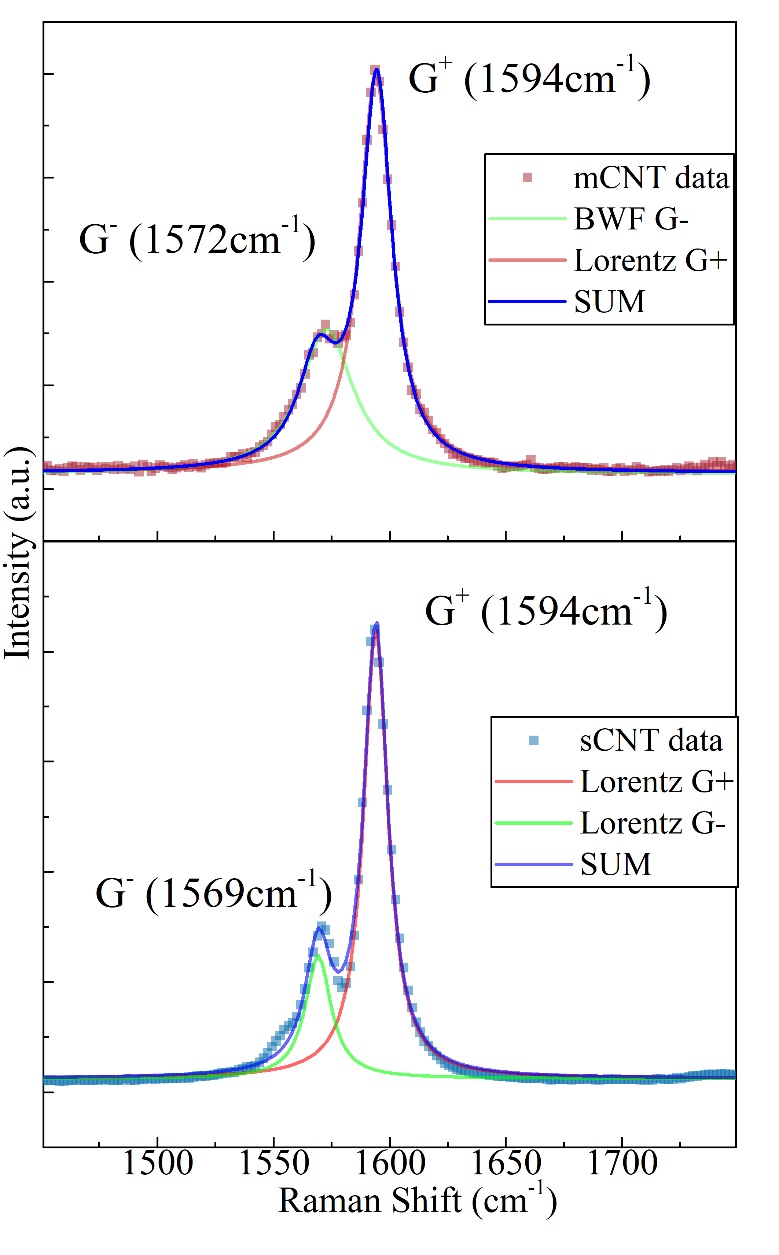 | 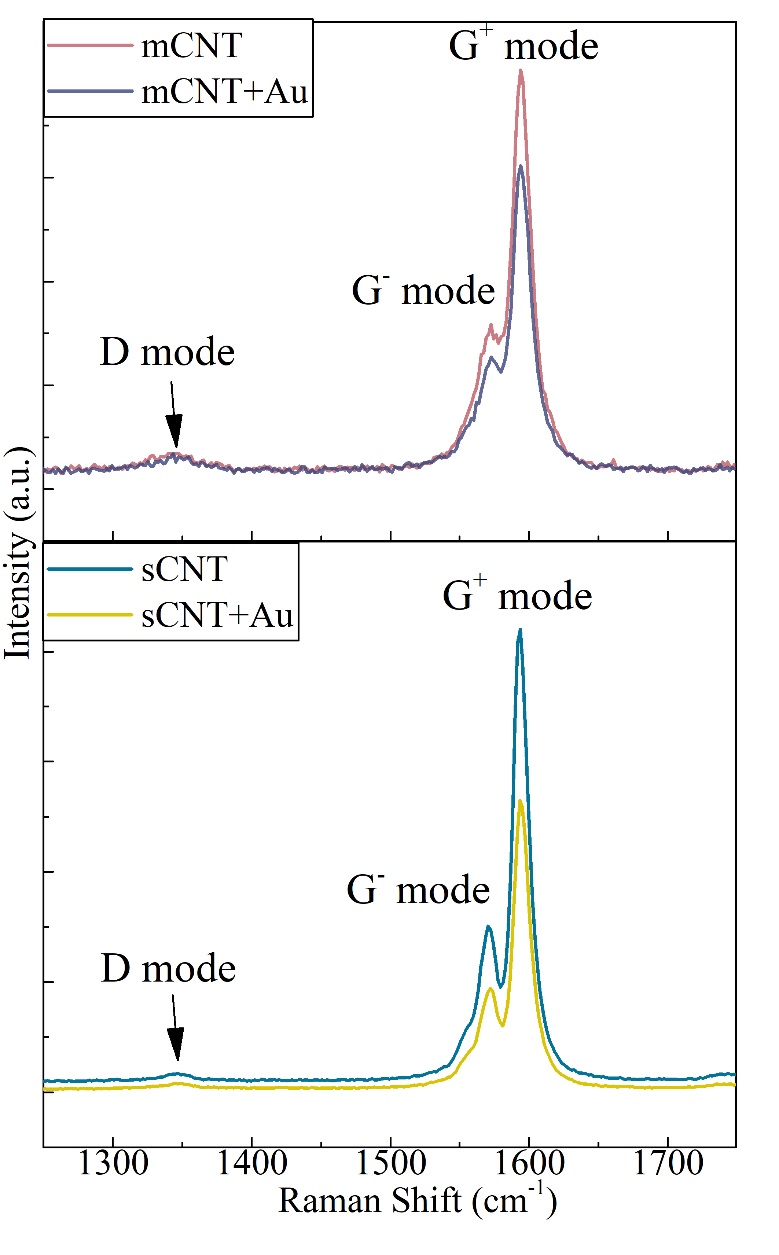 |
| --- | --- |

Figure S3 Left panel: The Lineshape analysis showing the differences between types of carbon nanotubes. The metallic CNTs G^-^mode are well described by Breigt – Winger -Fano (BWF) function. The semiconducting CNTs are well described by Lorenztian lineshapes (Lorentz). Right panel: All single spectra are characterized by low D mode intensity compare to G modes. This suggests the excellent quality of the investigated carbon nanotubes and the lack of the influence of the additional 10 min. sonication step for proper Au dots dispersion.

## **The statistical Raman shift analysis**

We performed Raman mapping on an area of 40 μm x 40 μm with a step of 4 μm. Next, for each spectra we have fitted Lorentzian or BWF function to obtain a set of parameters: positions of G^+^, G^-^, D, and 2D modes, which further has been used for correlation analysis in Figure 2 (in the main text) and for histograms in Figure S4. We used this approach in several of our previous works ^2,4–7^. The correlation from Figure 2 shows a general trend of the positions, but the magnitude of the position change has been obtained from the statistical distribution of the positions collected from Raman mapping. The shifts for metallic carbon nanotube thin film (Δω_G+_=0.14 cm^-1^, Δω_2D_ =0.24 cm^-1^) are lower compare to shift for semiconducting thin film(Δω_G+_=0.33 cm^-1^, Δω_2D_ =0.98 cm^-1^).

| 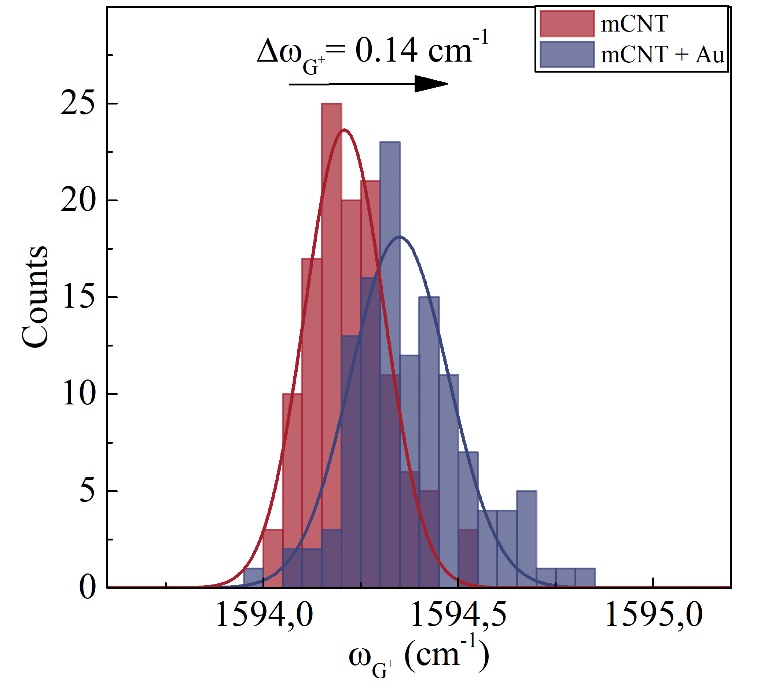 | 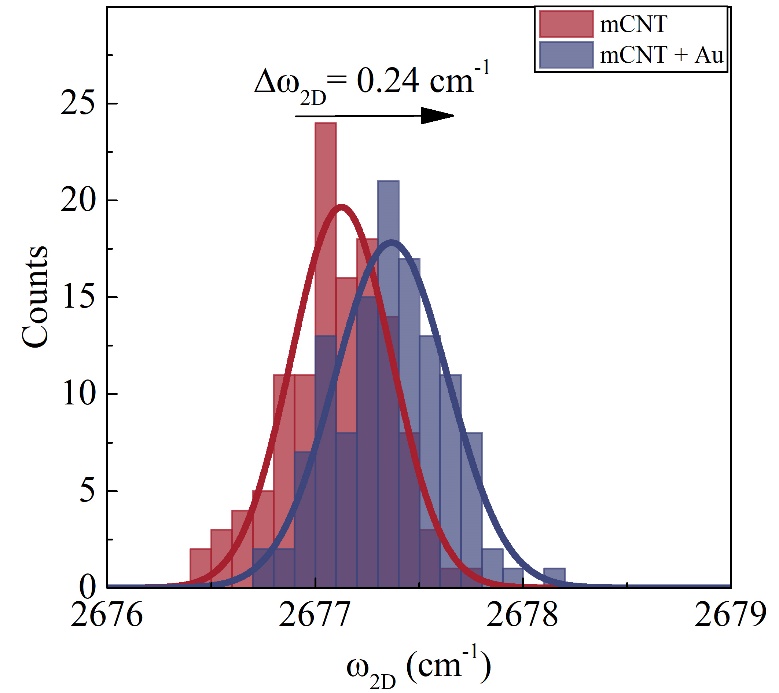 |
| --- | --- |
| 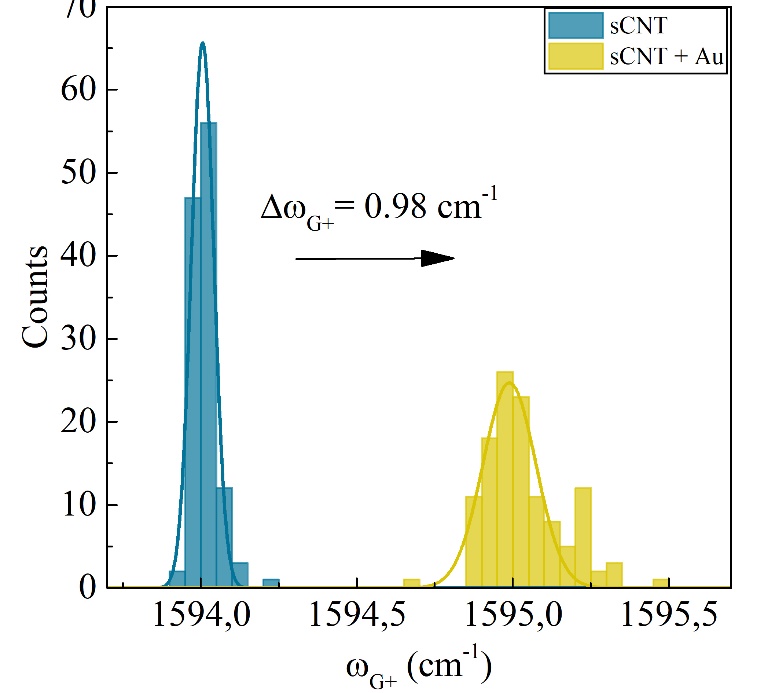 | 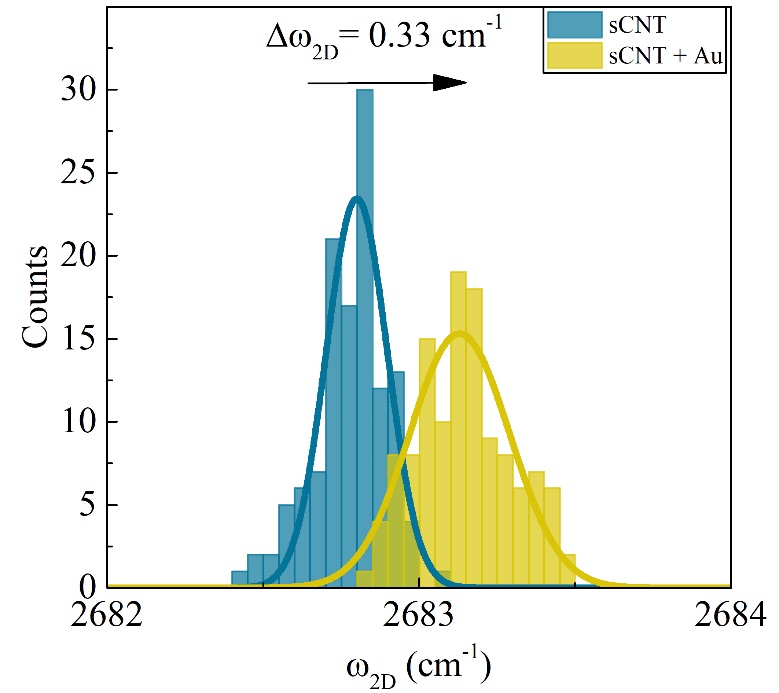 |

Figure S4 The histograms of the positions obtained from the fitting of the Raman map data. To histogram data, the Gauss distribution function has been fitted, and the position shift was the difference between fitted Gauss peak position.

# The de-doping and Au particles size influence on electrical measurements

Both phenomena: particle size and de-doping effect are influenced by high temperature annealing. Both phenomena have been widely investigated by Soo Min Kim *et.al.*. The de-doping effect, which is often related to the desorption of chloride anions from the ([AuCl_4_]^-^) in temperatures above 200 ^o^C^3^. We have annealed samples right after the production of thin films at 250 ^o^C for 2 h, to evaporates the MCE residues and most of the chloride anions. This step led to the creation of gold clusters, where nanodots were agglomerated (Figure S2). The high temperature annealing should make our electrical measurements stable with no further influence of Au nanoparticles' size change. The maximum temperature during electrical measurements was 450 K (177 ^o^C). Additionally, the work done by Teresa M. Barnes *et.al.* suggests that de-doping (reduction in doping effect) is associated with desorption of the dopants, and it was recorded only for the first trace^8^. Thus, the sheet resistance dependence on temperature is irreversible. In our case, we have observed a possible de-doping effect during the very first trace, which was used to reach the annealing temperature 450 K (Figure S5). Next, the annealing was performed for 12 h to avoid the de-doping influence caused by humidity contamination. This treatment leads to stable temperature traces, which have been characterized by negligible hysteresis (Figure S5 c, d).

| (a)  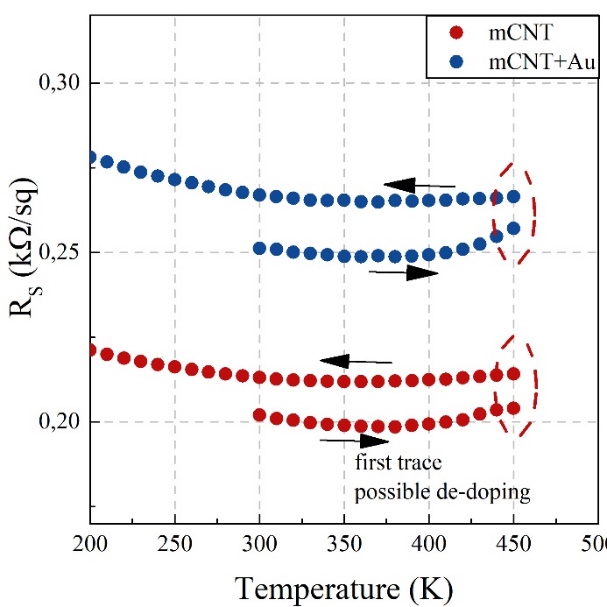 | (b)  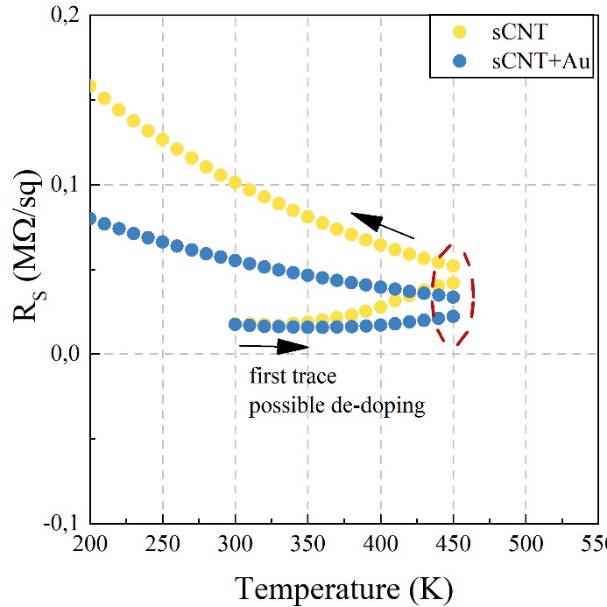 |
| --- | --- |
| (c)  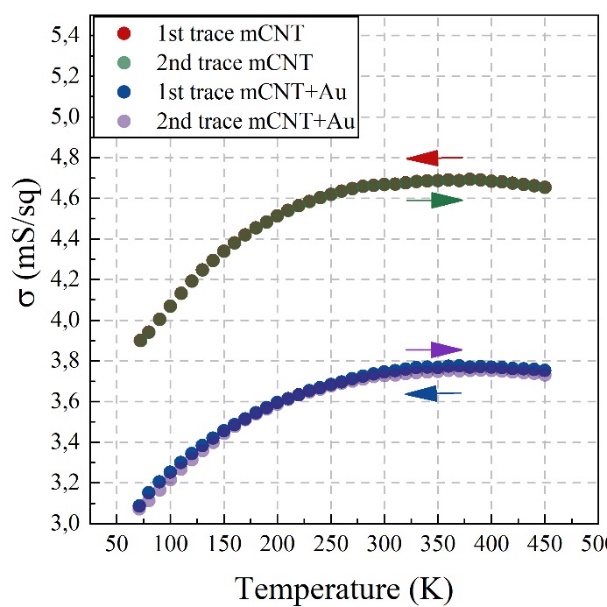 | (d)  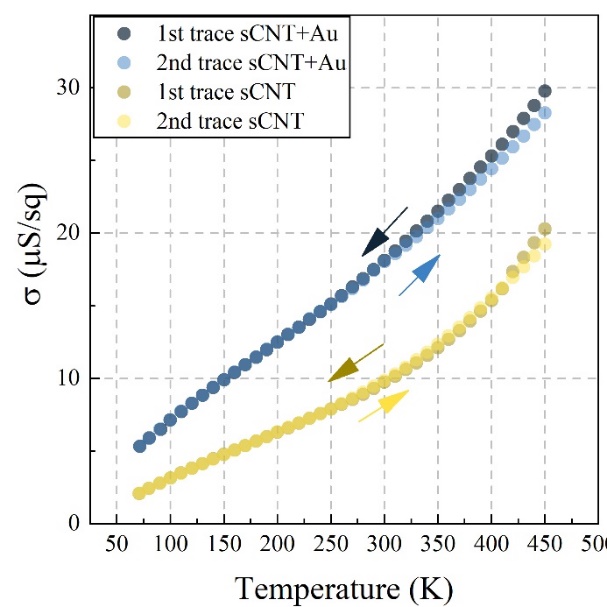 |

Figure S5 (a) and (b) The possible influence in de-doping for the very first trace before annealing for further electrical measurements. The marked areas show the difference in conductivities after 12 hours of annealing in a vacuum before main measurements. The values are slightly higher, which could be the progression in de-doping caused by humidity contamination. (c) and (d) both conductivity vs. temperature traces showing the negligible hysteresis and proves the electrical stability of our samples.

# The data collection and error discussion

We took a lot of attention to the accuracy of the data. We have created four contacts at the edges of the samples. Let’s mark them as A, B, C, D.

1. Starting from the van der Pauw measurements, we have collected data according to this method by measuring the I-V characteristics (Figure S6 a and b) of each combination of vdP measurement. From each I-V trace (5 points) we fitted the line and took slope as a value of Resistances (R_AB,CD_, R_BC,DA_ R_CD,AB_, R_DA,BC_), and their uncertainties:

$R_{AB,CD}=\frac{V_{D}-V_{C}}{I_{AB}}, R_{BC,DA}=\frac{V_{A}-V_{D}}{I_{BC}}$ $R_{CD,AB}=\frac{V_{B}-V_{A}}{I_{CD}}$,$R_{DA,BC}=\frac{V_{C}-V_{B}}{I_{DA}}$ (eq. 1)

This step has been done in LabView 2017 (National Instruments, Austin, TX, USA) software using its algorithm of the linear fitting.

1. Collected values of resistances and their uncertainties were further used to implement them into vdP function and its minimization:

$\exp\left( \frac{{-\pi R}_{A}}{R_{S}} \right)+\exp\left( \frac{{-\pi R}_{B}}{R_{S}} \right)=1$ ,(eq.2)

where $R_{A}=\frac{R_{AB,CD}+R_{CD,AB}}{2}, R_{B}=\frac{R_{BC,DA}+R_{DA,BC}}{2}$. Then equation 2 was solved numerically, obtaining the value of the sheet resistance Rs. The values were transformed into conductivity by 1/R_s_. The set of conductivity and temperature were used for further analysis.

1. To obtain the uncertainty of R_S_, u(R_S_) we propagate the uncertainty of measured R_AB,CD_, R_BC,DA_ R_CD,AB_, R_DA,BC_ by solving eq. 2 with values R ± u(R).
2. The value had been propagated onto conductivity using the Error Propagation Method:

$u\left( \sigma\right)=\sqrt{\left( \frac{\partial\left( \frac{1}{R_{S}} \right)}{\partial R_{H}}u(R_{S}) \right)^{2}}$, (eq.3)

The obtained values of uncertainty are below 1% for both mCNT and sCNT samples. We present below how it looks like on the single trace for metallic and semiconducting CNTs. We would like to highlight that error bars for obtained traces are almost not visible.

| 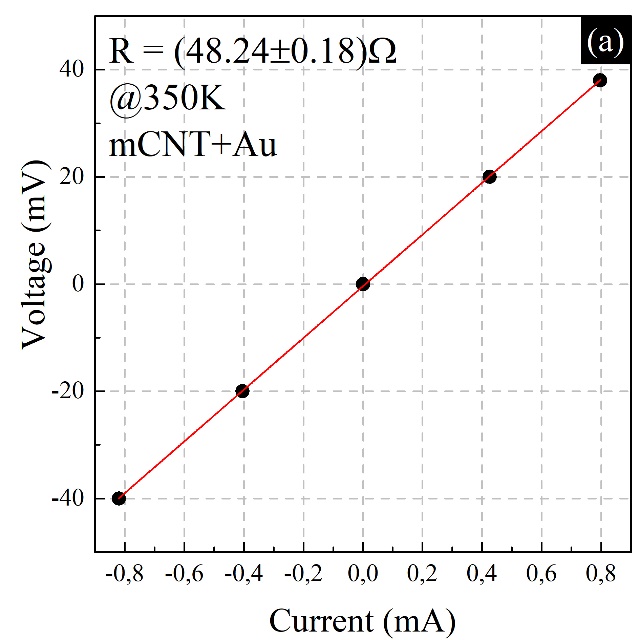 | 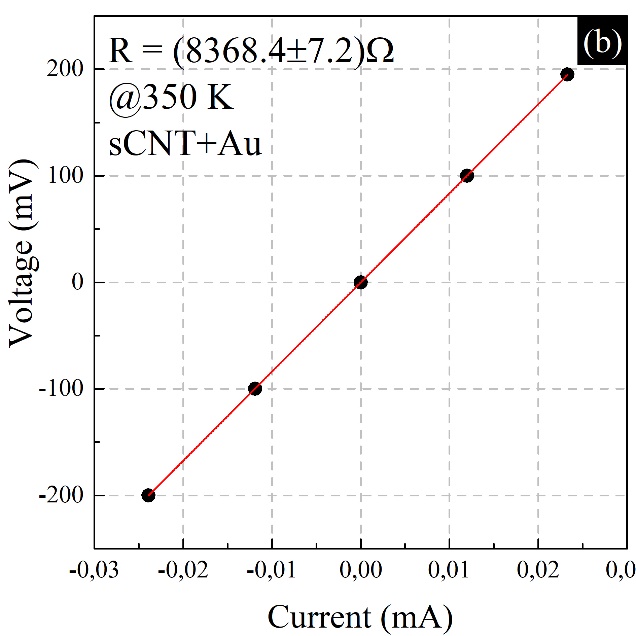 |
| --- | --- |
| 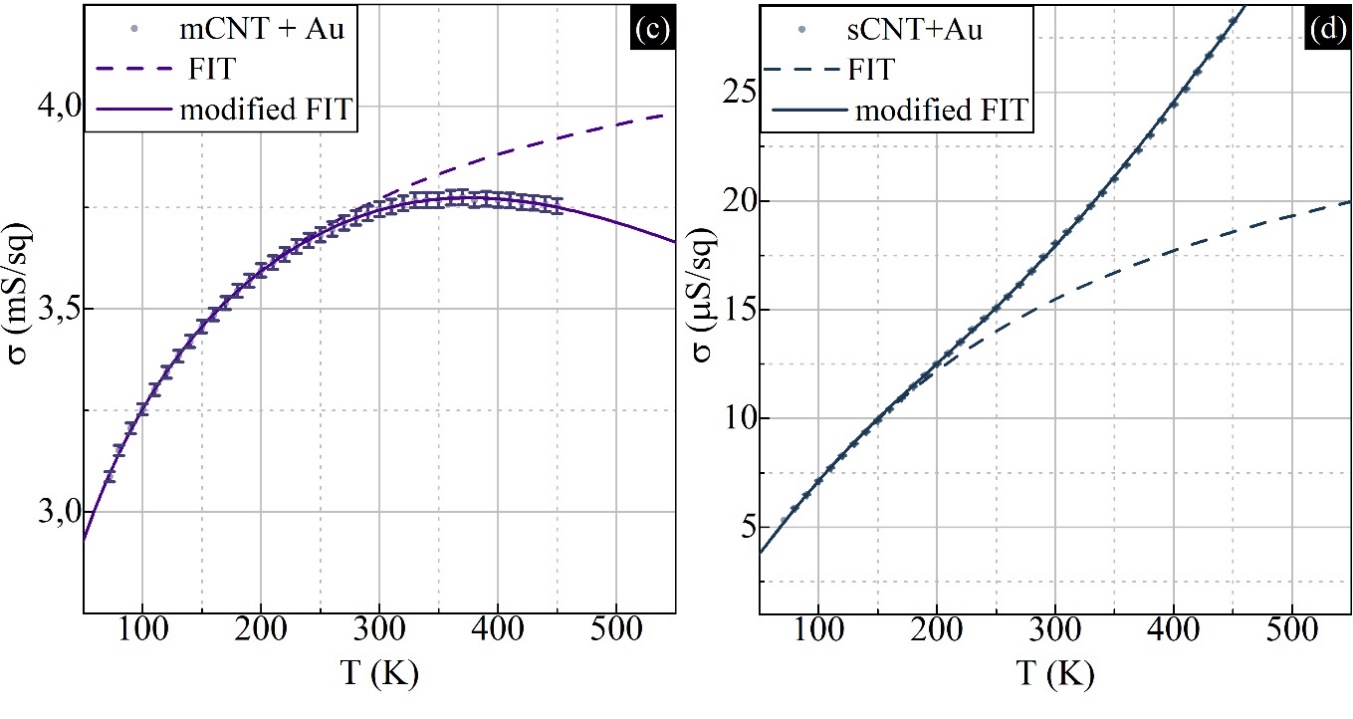 | |

Figure S6 (a) and (b) examples of collected V-I traces from the automated measurements in LabView. The red line is the linear fitting performed in Origin Pro software^9^ for utilization in the error discussion section. (c) and (d) the single traces of conductivity with error bars obtained from testing the Solver algorithm. The Error bars are around 0,4% of the main value.

We would like to also mention, that to minimize uncertainty involving temperature dependent study, our measurements setup has been automatized and self-stabilized during the temperature traces. To get the most accurate values, we programmed the stabilization based on (i) temperature stabilization with Mercury iTC controller and (ii) sample resistance fluctuation. The temperature stabilization was performed by a feedback loop of constant reading temperature, temperature fluctuation should be below 0.1K. The resistance control has been performed identically, the fluctuation of resistances has been kept below 1% of the measured value. When both conditions have been satisfied, the setup waited an additional 5 min before the measurement. The measurement was taken twice for each value of resistances.

## **The determination of the electrical transport model**

The Variable Range Hopping formula is described below:

$\sigma^{-1}\left( T \right)=\rho(T)=\left( A exp\left[ \left( \frac{T_{0}}{T} \right)^{\gamma} \right]+B exp\left[ \pm\frac{E_{a}}{k_{B}T} \right] \right)^{-1}$, (S1)

where γ describes model type: γ=1/2 refers to Efros-Shklvoski model^10^, γ=1/3, 1/4 refers to 2D and 3D VRH model, respectively. The “Mott energy” T_0_ is related to the density of localized states. The “E_a_” refers to two additional formulas, “-E_a_” for phonon scattering (1D transport along CNT) in mCNT and “+E_a_“for band-like transport in sCNT.

The four common transport models in the disordered system have been fitted to all four of our samples. Figure S3 presents all fitted data with additional comparison graphs. The comparison graphs present the ratio of the experimental data to the fitted model. We have also marked the red dashed area as a guide for an eye to see where the theory deviates from the experimental data. It is worth to mention, that if we would like to determine the transport model just by looking at the chart without any additional analysis, we could claim that every transport model is in reasonable agreement with the measured data. We would like to underline the fact, that with this additional analysis, we choose the modified-FIT model as the predominant transport model. For all presented data, the modified-FIT model shows the smallest difference between experimental and fitted data.


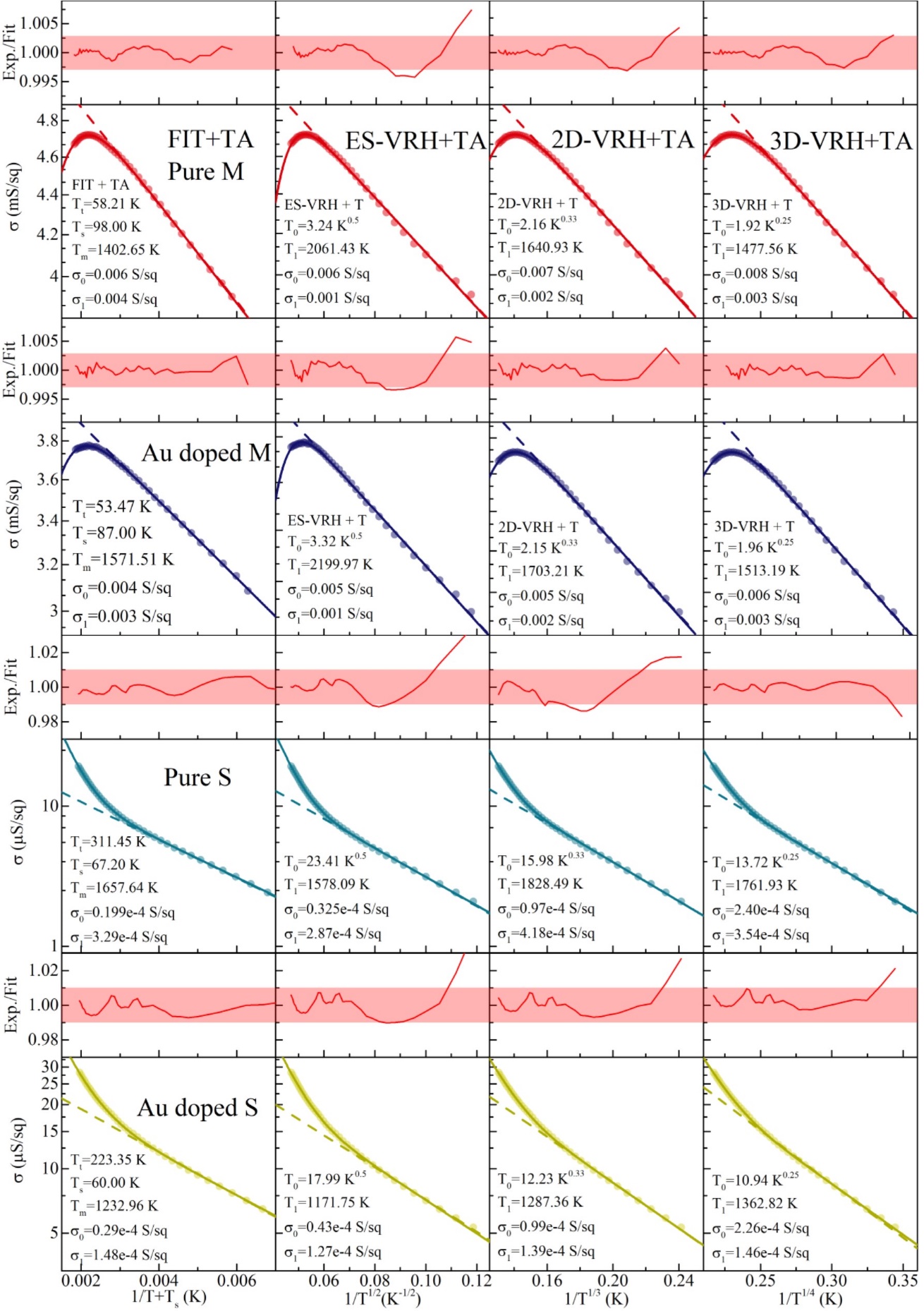


Figure S7 The comparison of the electronic transport models for FIT, 3D-VRH, 2D-VRH, and ES-VRH. The solid lines represent fitted modified transport models with temperature activated (TA) factors. The dashed lines present non-modified models. Pure M and S refer to undoped carbon nanotubes thin-film metallic and semiconducting, respectively. Au doped M and S refer to doped carbon nanotube thin-film metallic and semiconducting, respectively.

## **References**

1. Duzynska, A. *et al.* Phonon properties in different types of single-walled carbon nanotube thin films probed by Raman spectroscopy. *Carbon N. Y.* **105**, 377–386 (2016) doi:10.1016/j.carbon.2016.04.064.

2. Wroblewska, A., Gordeev, G., Duzynska, A., Reich, S. & Zdrojek, M. Doping and plasmonic Raman enhancement in hybrid single walled carbon nanotubes films with embedded gold nanoparticles. (2021) doi:10.1016/j.carbon.2021.04.079.

3. Kim, S. M. *et al.* Role of Anions in the AuCl 3 -Doping of Carbon Nanotubes. *ACS Nano* **5**, 1236–1242 (2011) doi:10.1021/nn1028532.

4. Wróblewska, A. *et al.* Statistical analysis of the reduction process of graphene oxide probed by Raman spectroscopy mapping. *J. Phys. Condens. Matter* **29**, 475201 (2017) doi:10.1088/1361-648x/aa92fe.

5. Judek, J. *et al.* High accuracy determination of the thermal properties of supported 2D materials. *Sci. Rep.* **5**, 12422 (2015) doi:10.1038/srep12422.

6. Gertych, A. *et al.* Complexity of temperature-dependent Raman spectra and phonons properties on the example of carbon nanotubes thin films. (2020) doi:10.1002/jrs.5930.

7. Świniarski, M., Wróblewska, A., Dużyńska, A., Zdrojek, M. & Judek, J. Kinetics of the thermal reduction process in graphene oxide thin films from in-situ transport measurements. *Mater. Res. Express* **8**, 015601 (2021) doi:10.1088/2053-1591/abdc50.

8. Barnes, T. M., Blackburn, J. L., van de Lagemaat, J., Coutts, T. J. & Heben, M. J. Reversibility, Dopant Desorption, and Tunneling in the Temperature-Dependent Conductivity of Type-Separated, Conductive Carbon Nanotube Networks. *ACS Nano* **2**, 1968–1976 (2008) doi:10.1021/nn800194u.

9. Origin(Pro), Version 2019b. OriginLab Corporation, Northampton, MA, USA.

10. Efros, A. L. & Shklovskii, B. I. Coulomb gap and low temperature conductivity of disordered systems. *J. Phys. C Solid State Phys.* **8**, L49–L51 (1975) doi:10.1088/0022-3719/8/4/003.
